# CT guided percutaneous needle biopsy of the chest: initial experience

**DOI:** 10.11604/pamj.2016.23.211.7865

**Published:** 2016-04-21

**Authors:** Younes Lazguet, Rachid Maarouf, Marouan Karrou, Imane Skiker, Ihsan Alloubi

**Affiliations:** 1 Department of Radiology, University Hosiptal Mohammed VI, Oujda, Morocco; 2 Department of Thoracic Surgery, University Hosiptal Mohammed VI, Oujda, Morocco

**Keywords:** Percutaneous biopsy, lung, CT

## Abstract

The objective of this article is to report our first experience of CT guided percutaneous thoracic biopsy and to demonstrate the accuracy and safety of this procedure. This was a retrospective study of 28 CT-Guided Percutaneous Needle Biopsies of the Chest performed on 24 patients between November 2014 and April 2015. Diagnosis was achieved in 18 patients (75%), negative results were found in 3 patients (12,5%). Biopsy was repeated in these cases with two positive results. Complications were seen in 7 patients (29%), Hemoptysis in 5 patients (20%), Pneumothorax in 1 patient (4,1%) and vaso-vagal shock in 1 patient (4,1%). CT Guided Percutaneous Needle Biopsy of the Chest is a safe, minimally invasive procedure with high sensitivity, specificity and accuracy for diagnosis of lung lesions.

## Introduction

CT Guided Percutaneous Needle Biopsy of the Chest is a minimally invasive procedure frequently indicated for the diagnosis of lung lesions [1]. This technique was first described in 1976 [2] and CT guidance is the primary Imaging modality. Core needle biopsies provide high quality tissue samples for histologic analysis and immunohistochemical testing [3, 4]. Complications include pneumothorax, haemorrhage, air embolism and tumor seeding, with pneumothorax being the most frequent [5]. Throughout the world, this technique is useful and accurate in the diagnosis of peripheral masses. Our study demonstrates the accuracy of this technique, confirms its safety with low complication rates, especially with the use of co axial needles.

## Methods

**Figure 1:**
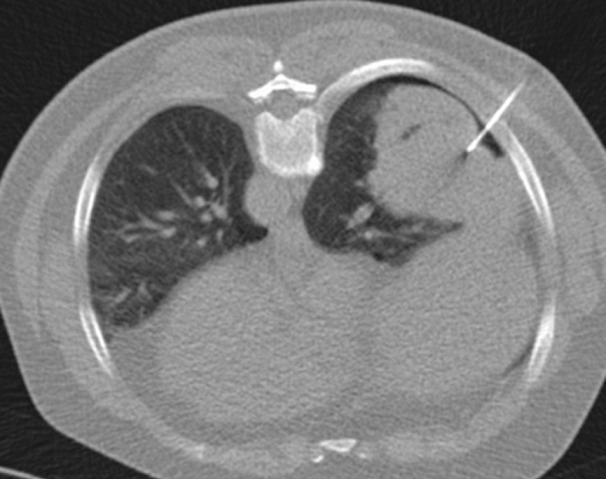
Axial CT image showing post procédural pneumothorax in 65 years old man who underwent core needle biopsy of right postéro basal mass

24 patients were included to this retrospective study; they were admitted to the Thoracic surgery department between October 2014 and April 2015. These patients underwent lung biopsies with a core needle biopsy device (16G or 18G) under CT guidance (SOMATOM Definition AS plus 128, Siemens Healthcare, Forchheim, Germany). All patients with peripheral masses suspicious for malignancy and without suspicion of vascular disease were included in this study. The patients were admitted one day before the procedure to the thoracic surgery department and a coagulation test was done (prothrombin time, activated prothrombin time and platelet count). One patient was on oral anticoagulants for atrial fibrillation (Coumadine*), the treatment was replaced with heparin 5 days before the procedure [6]. We informed patients about the benefits and risks of the procedure such as pneumothorax and haemorrhage and consent was obtained. A peripheral IV cannula was placed before installing patient in the CT scanner. All biopsies were done under CT guidance. The patient was positioned prone, supine or in lateral decubitus, according to the location of the lesion. The lesion was localised and the access route was planned using our Chest intervention protocol including a first 2 mm slice thickness acquisition used to localise the lesion, then the entry site was marked by a permanent marker, and distance between the skin surface and the lesion was measured. Then, the skin entry site and surrounding skin area was cleaned by povidone iodine solution and covered by sterile drapes. Local anaesthesia at the entry site and biopsy track was performed with 5 - 10 cc of Lidocaine 2%. A 17G, 11 cm co axial needle (Angiotech, Argon Medical*) was placed in the predetermined route and Advanced carefully Under CT guidance to verify its position using 5 mm sequential acquisitions to minimize radiation exposure. Once the coaxial needle was in satisfactory position, it was Advanced rapidly through the pleura and placed at the edge or directly within the lesion. The inner part of the coaxial needle was removed and an 18 G 16 cm tru-cut semi automatic needle was placed to obtain 3 or 4 samples. The samples were placed in formalin solution and sent to Histopathology for analysis. The core needle biopsy device was removed and the entry site was covered with sterile gauze, then 2 mm axial slices covering the biopsy level were acquired to check for complications. Then the patients were transferred to observation room and placed on the opposite side to the entry site for biopsy and observed for 2 hours. Patients with pneumothorax were placed under nasal oxygen and a Chest X-ray was done 24 hours later to check the evolution of the pneumothorax. No chest tubes were inserted into any of our patients (Figure 1)

## Results

24 patients (23 males and 1 female) were included in our study. The patients’ mean age was 62 years (43 - 90 years). 22 patients were active smokers. Mean lesion size was 43 mm and the median traversed parenchymal distance was 20 mm. 21 patients had peripheral lung lesions whereas 3 had central lesions. Lesions were solid in 10 patients, solid and necrotic in 11 patients and purely necrotic in 3 patients. Emphysema was seen in 10 patients and pneumothorax was sustained in 2 patients. A total of 27 biopsies were performed in 24 patients. Histopathology results were positive in 18 of 24 patients and negative in 3 patients who underwent a second biopsy. All had malignant lesions. 7 patients in our study developed complications (29,17%): Hemorrhage in 5 patients « 20, 8% » (3 mild and 1 moderate); small volume pneumothorax in 1 patient « 4,2% »; vasovagal shock in 1 patient « 4,2% ». Alveolar hemorrhage and pneumothorax resolved spontaneously with no need to proceed to other interventions. One patient who received anticoagulation for atrial fibrillation underwent biopsy. Their Warfarin was stopped 5 days before the procedure and replaced with heparin [6]. A small area of alveolar hemorrhage was seen on post-biopsy CT, without any associated clinical symptoms.

## Discussion

In our retrospective study on CT Guided Percutaneous Needle Biopsy, diagnostic accuracy rate was 75%. Non diagnostic biopsy rate was 12,5%. Complications were essentially alveolar hemorrhage (20, 8%) and pneumothorax (4,2%) in a smaller proportion. Spontaneous reabsorption of alveolar hemorrhage and pneumothorax was seen in all patients with no need to proceed to other interventions such as chest drain insertion. Percutaneous CT-guided core needle biopsy is a safe procedure with a high accuracy for diagnosis of lung malignancies [7]. Our results confirm that CT Guided Percutaneous Lung Biopsy is a safe procedure allowing diagnosis of lung lesions in the majority of patients. Major complications were pneumothorax and hemorrhage with good outcomes and spontaneous reabsorption. The presence of emphysema was challenging, meaning a greater risk of pneumothorax, most common in our smoking patient population [8]. Deeper lesions require traversing a greater amount of lung parenchyma and increase the risk of pneumothorax and hemorrhage; we did not notice any of those incidents in our study. We thought that small anterior and lateral lesions were highly challenging to reach because of respiratory motion; so good communication with the patient and soft respiration were mandatory to successfully achieve the procedure with few or no complications. Limitations of our study were the small number of patients included and the retrospective design.

## Conclusion

In conclusion, CT-guided percutaneous needle biopsy is a safe procedure with a high diagnostic accuracy rate in the diagnosis of malignant lung lesions.

### What is known about this topic

- Percutaneous thoracic CT guided biopsy is an accurate procedure for diagnosis of lung malignancies;
- Major complications are pneumothorax and alveolar hemorrhage;
- CT is a very helpful tool to localize the lesion and achieve the procedure.

### What this study adds

- Percutaneous thoracic CT guided biopsy with 18 G co axial needle is a very safe procedure with lesser complication rate;
- CT protocol used in this study limits the radiation exposure of patients;
- The procedure can also be achieved in emphysematous patients without increasing complication rate.
